# A Case of a Man Who Died from Eating to Excess of the Bitter Almond

**Published:** 1827-02

**Authors:** 


### DEATH FROM EATING BITTER ALMONDS.

*A Case of a Man who died from eating to excess of the Bitter Almond*, by Mr. Kennedy. *With some Remarks*, by Dr. Paris.

September, 1825, I was called to see a man stated to have fallen down dead in Trinity-street, Borough. I went immediately to the spot, and found the poor fellow laid upon timber, supported by some labourers. I directed him to be taken to the Roebuck Inn close by. At this time the pulse was scarcely perceptible at the wrist, and the extremities were quite cold. The account given by the person who first took notice of him (a master builder) was—that he saw a man, apparently in pain, endeavouring to make water against the wall, and as if he were sinking down: he called out to him; but the man took no notice, and in a minute afterwards fell. He ran to him, but neither heard a sigh nor groan.

On his being removed into the house, I attempted to bleed him from the jugular vein, and then from the arm: about an ounce of very dark-coloured blood flowed. He was laid upon a table, and there fell out of his pocket some bitter almonds: this led me to the conjecture that he had been poisoned by eating them; and I was further convinced of it by perceiving the peculiar odour of the hydrocyanic acid in his mouth. I then ordered him a glass of hot brandy and water: after this had been poured down his throat, a stream of blood flowed from his arm, which was still kept bound; and I thought I felt the heart beat, but I did not feel the pulse. Another glass of hot brandy and water was given, but life was now extinct. The eyes, which had been natural, were fixed but shining, (which appearance they retained for some time;) while foam and mucus issued from his mouth and nares.

The following morning, twenty hours post mortem, I made the examination in company with a friend, Mr. John Hill, surgeon. The body had more of a purplish hue than usual, particularly where any pressure had been applied,—as on the arm where the fillet had been bound, on the neck, &c. Mr. Hill placed his hand on the chest, and said "There is some degree of warmth here;" but I could not perceive it: however, another gentleman, (not of the profession,) then in the room, was of Mr. H.'s opinion.

I may remark here, that I had been before much surprised and puzzled to find the extremities cold, while much warmth remained about the chest. I ordered hot fomentation, which, of course, was of no avail; and more than four hours after, when I was fully convinced life was gone, I felt considerable warmth at the chest: he was laid in a shell, and in a cold room.

The large vessels of the brain were distended with blood, as in apoplexy, but the organ was otherwise sound and healthy in its substance. The heart and lungs were healthy. On opening the abdomen, and exposing the stomach, it was found immensely distended, and strongly smelling of the hydrocyanic acid. I removed it carefully, and, on laying it open, some gas escaped. A quantity (two pounds at least) of undigested matter was in it, amongst which many pieces of almonds could be distinctly seen: indeed, the mass appeared to consist of little else. No inflammation of the stomach, of the large or small intestines, could be traced. The bladder was filled with water.

On searching his person to discover his residence, his pockets were all found more or less filled with bitter almonds; he had not a halfpenny in them: he looked very poor. We had no hesitation, therefore, in concluding that this man had died from the effects of the essential oil, or prussic acid, contained in the almonds, having eaten a large quantity to allay the cravings of hunger. I accordingly gave my deposition before the magistrates at Union Hall to this effect.

The man was of the middle size, strong and muscular; aged about forty years; his name and residence unknown.

Dr. Paris, in a letter to me on this subject, says, "There cannot be a doubt but that the death of the person was occasioned by the poison of the almond, although it may perhaps be said that the distention of the stomach by so undigestible an aliment might have occasioned congestion in the brain, and thus produced death. I am, however, of a different opinion. The warmth about the chest has been frequently observed in cases of narcotic poisoning. You have not stated distinctly whether the eyes retained their lustre; a fact which I consider as decisive evidence of poisoning by hydrocyanic acid. Mr. Earle informed me that, in the case of a professional man who had destroyed himself by the prussic acid, the glistening of the eye was so remarkable, that he almost hesitated whether he should proceed to the dissection."

I would observe, that some difference may be expected to be found in the appearance of different individuals, occasioned by the quantity as well as the quality of the poison taken; and I have lately heard of a case which strongly illustrates this, related by a friend, in which a considerable quantity of the hydrocyanic acid of the shops was taken, occasioning instant death, and yet, on dissection, the odour of the poison was not perceived in the stomach, but the coats were in a state of high inflammation.

